# Choroidal congestion mouse model: Could it serve as a pachychoroid model?

**DOI:** 10.1371/journal.pone.0246115

**Published:** 2021-01-28

**Authors:** Hidetaka Matsumoto, Ryo Mukai, Junki Hoshino, Mai Oda, Toshiyuki Matsuzaki, Yasuki Ishizaki, Koji Shibasaki, Hideo Akiyama

**Affiliations:** 1 Department of Ophthalmology, Gunma University Graduate School of Medicine, Maebashi, Japan; 2 Department of Molecular and Cellular Neurobiology, Gunma University Graduate School of Medicine, Maebashi, Japan; 3 Department of Anatomy and Cell Biology, Gunma University Graduate School of Medicine, Maebashi, Japan; 4 Laboratory of Neurochemistry, Graduate School of Human Health Science, University of Nagasaki, Nagasaki, Japan; Massachusetts Eye & Ear Infirmary, Harvard Medical School, UNITED STATES

## Abstract

Pachychoroid spectrum diseases have been described as a new clinical entity within the spectrum of macular disorders. “Pachychoroid” is defined as choroidal thickening associated with dilated outer choroidal vessels often showing retinal pigment epithelium (RPE) degeneration. Although various clinical studies on the pachychoroid spectrum diseases have been conducted, the pathophysiology of pachychoroid has yet to be fully elucidated. In this study, we attempted to establish a mouse model of pachychoroid. We sutured vortex veins in eyes of wild type mice to imitate the vortex vein congestion in pachychoroid spectrum diseases. Fundus photography and ultra-widefield indocyanine green angiography showed dilated vortex veins from the posterior pole to the ampulla in eyes after induction of choroidal congestion. Optical coherence tomography and tissue sections presented choroidal thickening with dilatation of choroidal vessels. The RPE-choroid/retina thickness ratios on the tissue sections in the treated day 1 and day 7 groups were significantly greater than that in the control group (0.19±0.03 and 0.16±0.01 vs. 0.12±0.02, P<0.05 each). Moreover, immunohistochemistry using RPE flatmount revealed focal RPE degeneration in the treated eyes. Furthermore, inflammatory response-related genes were upregulated in eyes with choroidal congestion induction, and macrophages migrated into the thickened choroid. These results indicated that vortex vein congestion triggered some pachychoroid features. Thus, we have established a choroidal congestion mouse model by suturing vortex veins, which would potentially be useful for investigating the pathophysiology of pachychoroid spectrum diseases.

## Introduction

Functional improvement of optical coherence tomography (OCT) has disclosed the existence of pachychoroid, i.e. choroidal thickening associated with outer choroidal vessels [1]. Several pachychoroid spectrum diseases including central serous chorioretinopathy (CSC) [2], pachychoroid pigment epitheliopathy (PPE) [3], pachychoroid neovasculopathy (PNV) [4], and polypoidal choroidal vasculopathy (PCV) [5] have been reported. Recent studies have suggested the development of neovascular age-related macular degeneration (AMD) in Asian patients to be strongly related to pachychoroid [6]. In terms of treating neovascular AMD, intravitreal injection of anti-vascular endothelial growth factor (VEGF) has been the first-line therapy based on the results of randomized clinical trials conducted in the United States [7, 8]. However, many studies in Asian countries have demonstrated the efficacy of photodynamic therapy for pachychoroid spectrum diseases [9–11]. Therefore, the pathophysiology underlying pachychoroid needs to be elucidated to understand the pathophysiology of neovascular AMD and its optimal treatments.

To date, various clinical studies on pachychoroid spectrum diseases have focused on genetics [6, 12, 13], the cytokine concentrations in aqueous humor [14, 15], OCT image analysis [16, 17], and the efficacy of treatments [11, 18]. However, the pathophysiology of pachychoroid spectrum diseases has yet to be fully elucidated. Many animal models of retinal diseases, including retinopathy of prematurity, retinal detachment, and glaucoma, have been established [19–21]. These models contribute to elucidating the underlying pathophysiological mechanisms and to the development of new treatments. Moreover, disease models using mice allow investigators to take advantage of the genetic manipulation which is possible in mice. An animal model of pachychoroid is needed to elucidate the pathophysiological mechanisms underlying pachychoroid spectrum diseases. Therefore, this study aimed to establish a mouse model of pachychoroid.

Many studies using multimodal imaging have examined the causes of pachychoroid spectrum diseases. In CSC, ultra-widefield indocyanine green angiography (ICGA) showed dilated choroidal vessels, the so-called pachyvessels, from the posterior pole to the ampulla of the vortex veins [22]. Moreover, the area of filling delay on ICGA markedly overlapped with the area of pachyvessels in CSC [16]. Our previous studies revealed that the anastomosis between superior and inferior vortex veins involving the macular area was more frequently observed in pachychoroid spectrum diseases than in eyes of normal subjects [17, 23]. In PNV and PCV, choroidal neovascularization was reportedly present at the site where the inner choroid was thinned due to the dilated outer choroidal vessels (pachyvessels) [5, 17]. Furthermore, a recent study using anterior-segment OCT showed scleral thickness to be greater in CSC than in normal eyes, although there were no differences in either spherical equivalent or axial length [24]. These reports suggest that the major cause of pachychoroid spectrum diseases might be vortex vein congestion.

In the current study, we attempted to establish a mouse pachychoroid model by suturing the vortex veins at the scleral surface to imitate the vortex vein congestion in pachychoroid spectrum diseases.

## Materials and methods

### Animals

All animal procedures were performed in accordance with the ARVO Statement for the use of Animals in Ophthalmic and Vision Research and the National Institutes of Health Guidance for Care and Use of Laboratory animals. The protocol was approved by the Gunma University Ethics Committee. We used 3 BALB/c and 35 C57BL/6 mice in total for this study (Japan SLC, Hamamatsu, Shizuoka, Japan). The choroidal congestion was induced in the right eye. The left eye was used as a control, in which the conjunctiva was not incised. Three BALB/c mice were used for fundus photography 1 day after the induction of choroidal congestion. 3 and 2 C57BL/6 mice were used for ultra-widefield ICGA and DNA microarray, respectively, 1 day after the treatment. 4, 6, and 3 C57BL/6 mice were used to obtain OCT images, cryosections, and resin-embedded sections, respectively, at both 1 and 7 days after the choroidal congestion induction. Four C57BL/6 mice were used for RPE flatmount 7 days after the treatment. All mice were male with age 10±2 weeks, whereas the age distributions in control and treated groups were same in each experiment.

### Induction of choroidal congestion

**Fig 1:**
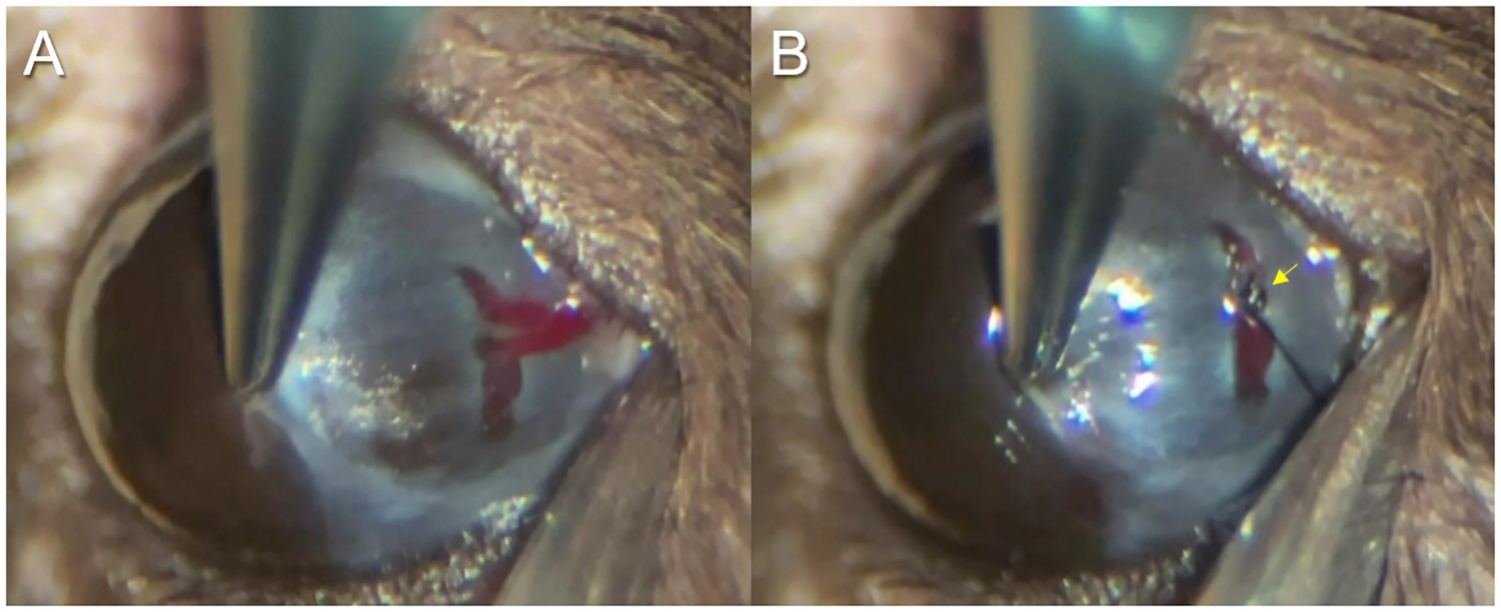
Induction of choroidal congestion in the mouse eye. (A) After the conjunctiva in the inferotemporal quadrant had been incised and separated from the sclera, two vortex veins were observed at the surface of the sclera. (B) The blood flow to the venous sinus was stopped after suturing the vortex veins with 10–0 nylon surgical suture (yellow arrow). All vortex veins in each quadrant of the right eye were sutured to induce choroidal congestion.

Mice were anesthetized with an intraperitoneal injection of 50 mg/kg Nembutal. The conjunctiva at each quadrant was incised and detached from the sclera. 1 or 2 vortex veins are observed in each quadrant of the mouse eye (Fig 1A). All vortex veins of the right eye were sutured at the surface of the sclera employing 10–0 nylon surgical suture (MANI, INC., Utsunomiya, Tochigi, Japan), aiming to induce vortex vein congestion (Fig 1B and S1 Video). All surgeries were performed by the first author.

### Ultra-widefield indocyanine green angiography

Mice were anesthetized with an intraperitoneal injection of 50 mg/kg Nembutal, and the pupils were dilated with topical drops of a phenylephrine (5%) and tropicamide (0.5%) mixture. After intraperitoneal injection of 2 mg/kg ICG, images were captured by an Optos ultra-wide-field retinal imaging device equipped with a light source with an 802 nm emission wavelength (California; Dunfermline Scotland, UK). Mice were placed on the device, such that the fundus was in focus. Fundus images were taken without any additional contact or non-contact lenses.

### Optical coherence tomography

Mice were anesthetized with an intraperitoneal injection of 50 mg/kg Nembutal, and the pupils were dilated with topical drops of a phenylephrine (5%) and tropicamide (0.5%) mixture. OCT B-scan images each composed of the 20 averaged scans using eye tracking were obtained from the mid-periphery of the inferior fundus (Spectralis HRA + OCT; Heidelberg Engineering, Heidelberg, Germany). Mice were placed on the device, such that the fundus was in focus. OCT images were taken with a non-contact lens attached to the device.

### Resin-embedded sectioning and toluidine blue staining

Mice were anesthetized with an intraperitoneal injection of 50 mg/kg Nembutal, and perfused with a mixture of 2% paraformaldehyde and 2.5% glutaraldehyde in 0.1 M phosphate buffer (pH 7.4) followed by physiological saline. Eyes were enucleated and their anterior segments including the cornea, iris, and lens were removed under a microscope. The eye cups were incubated in the same fixative overnight. After washing with phosphate buffered saline (PBS), the eye cups were post-fixed with 1% OsO4 in 0.1 M phosphate buffer (pH 7.4) for 1 hour, washed with pure water, and then dehydrated through a graded ethanol series. The eye cups were placed in propylene oxide for 30 min, infiltrated with increasing concentrations of Quetol-812 epoxy resin mixed with propylene oxide overnight and finally embedded in pure Quetol-812 epoxy resin. Semi-thin sections from resin-embedded tissues were cut at a 0.5-μm thickness on an ultramicrotome (ULTRACUT UCT; Leica), placed on glass slides, and stained with 0.5% toluidine blue in 0.5% sodium borate solution for light microscopy.

### Cryosection and measurement of RPE-choroid/retina thickness ratio

Eyes were enucleated and embedded in OCT compound (Tissue-Tek; Sakura Finetek Japan Co., Tokyo, Japan) without fixation. Serial sections in the sagittal plane were cut at a 10-μm thickness on a cryostat (CM3050 S; Leica) at -20 °C. The RPE-choroid/retina thickness ratio was measured on the cryosection showing the center of the optic nerve. The ratio was measured at 600μm from the margin of the optic nerve (S1 Fig). The average of RPE-choroid/retina thickness ratios on both sides of the optic nerve was determined as the RPE-choroid/retina thickness ratio of the eye. The Steel-Dwass test was used for multiple analyses comparing RPE-choroid/retina thickness ratios in control, treated day 1, and treated day 7 eyes. The data analysis was performed using Excel 2016 (Microsoft, Redmond, WA, USA) with add-in software Statcel4 [25]. A P<0.05 was considered to indicate a statistically significant difference. All values are presented as the average ± standard deviation.

### Immunohistochemistry

Cryosections were fixed in 4% paraformaldehyde for 5 minutes, blocked in 3% bovine serum albumin in PBS containing 0.3% Triton X-100 for 20 minutes, and incubated with rat anti-CD11b antibody (1:50; catalog number 550282, BD Biosciences, San Jose, CA, USA) at room temperature for 2 hours. AlexaFluor 488–conjugated goat anti-rat IgG was used as a secondary antibody and the sections were incubated at room temperature for 30 minutes. Finally, the sections were counterstained with DAPI. Fluorescence images were captured using a confocal microscope (LSM880, Zeiss Japan, Tokyo, Japan).

### Flatmount staining

Eyes were enucleated and fixed in 4% paraformaldehyde for 1 h. After washing with PBS, the anterior segment and the neuroretina were removed under a microscope. The RPE–choroid–sclera complex was blocked with 3% bovine serum albumin in PBS containing 0.3% Triton X-100 for 1 h and was incubated with FITC-conjugated ZO-1 antibody (1:100; catalog number 33–9111, Invitrogen, Camarillo, CA, USA) at room temperature for 3 hours. Fluorescence images were captured using a fluorescence microscope (BZ-9000, Keyence, Osaka, Japan).

### Total RNA isolation

Right after enucleation of eyes, the anterior segment and the neuroretina were removed under a microscope. Then, the RPE–choroid–sclera complex was immediately homogenized using a glass homogenizer. Total RNA was isolated employing the RNeasy Mini Kit (QIAGEN, Valencia, CA, USA) according to the manufacturer’s protocol.

### cRNA amplification and labeling

RNA quantity and quality were determined using a Nanodrop One spectrophotometer (Thermo Fisher Scientific Inc., Waltham, MA, USA). We assessed total RNA quality by 260 nm/280 nm absorbance, and only utilized samples that had a 260 nm/280 nm ratio >2.0. Furthermore, we confirmed the RNA quality with an Agilent Bioanalyzer (Agilent Technologies, Santa Clara, CA, USA), as per the manufacturer’s protocol. Total RNA was amplified and labeled with Cyanine 3 (Cy3) using an Agilent Low Input Quick Amp Labeling Kit, one-color (Agilent Technologies) following the manufacturer’s instructions. Briefly, total RNA was reversed transcribed to double-stranded cDNA using a poly dT-T7 promoter primer. Primer, template RNA and quality-control transcripts of known concentration and quality were first denatured at 65 °C for 10 min and incubated for 2 hours at 40 °C with 5X first strand Buffer, 0.1 M DTT, 10 mM dNTP mix, and AffinityScript RNase Block Mix. The AffinityScript enzyme was inactivated at 70 °C for 15 min. cDNA products were then used as templates for in vitro transcription to generate fluorescent cRNA. cDNA products were mixed with a transcription master mix in the presence of T7 RNA polymerase and Cy3 labeled-CTP and incubated at 40 °C for 2 hours. Labeled cRNAs were purified using QIAGEN’s RNeasy mini spin columns and eluted in 30 μl of nuclease-free water. After amplification and labeling, cRNA quantity and cyanine incorporation were determined using a Nanodrop One spectrophotometer and an Agilent Bioanalyzer. For each sample hybridization, 0.60 μg of Cy3 labeled cRNA were fragmented, and hybridized at 65 °C for 17 hours to an Agilent SurePrint G3 Mouse Gene Expression 8x60K version 2.0 (Design ID: 074809). After washing, the microarrays were scanned using an Agilent SureScan Microarray Scanner System (G4900DA). The array had 56,605 probes.

### Data analysis of microarray

Intensity values of each scanned feature were quantified using Agilent feature extraction software version 12.1.1.1, which performs background subtractions. We only used features which were flagged as no errors (Detected flags) and excluded features which were not positive, not significant, not uniform, not above background, saturated, and/or population outliers (Not Detected and Compromised flags). Normalization was performed by percentile shift to the 75th percentile using Agilent GeneSpring. We calculated the average expression values for each gene in control and treated groups, and analyzed the temporal changes. Then, we designated genes showing an over 1.5-fold change as increased genes, and those showing an under -1.5-fold change as decreased genes.

## Results

**Fig 2:**
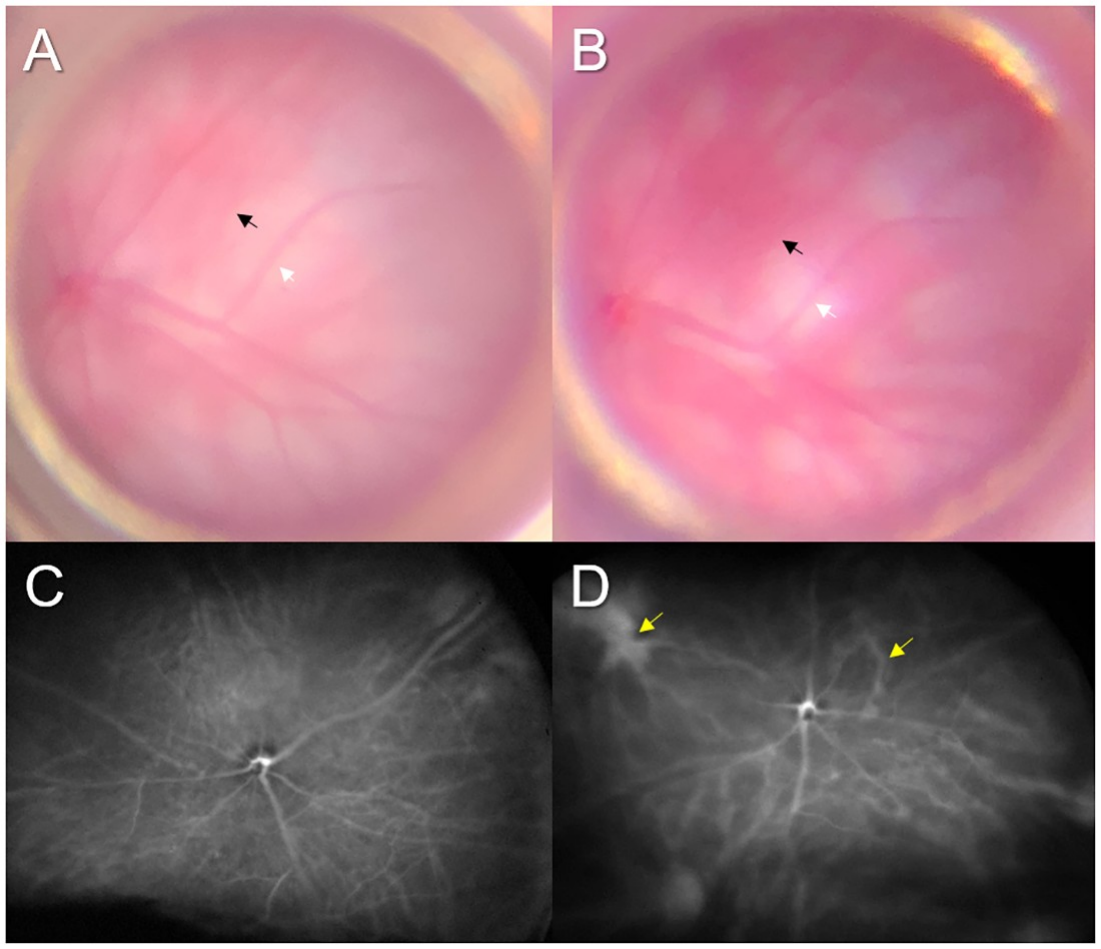
Dilatation of choroidal vessels after suturing of the vortex veins. (A) Fundus photograph of BALB/c mouse eye shows not only retinal vessels (white arrow) but also choroidal vessels (black arrow) because of the non-pigmented retinal pigment epithelium. (B) 1 day after suturing of the vortex veins, dilated choroidal vessels (black arrow) can be seen whereas the retinal vessels (white arrow) are unchanged. (C) Ultra-widefield ICGA in the untreated C57BL/6 mouse eye shows normal retinochoroidal vessels. (D) 1 day after suturing of the vortex veins, dilated vortex veins can be seen from the posterior pole to the ampulla of vortex veins (yellow arrows) despite the normal appearance of retinal vessels.

In our preliminary experiments, we sutured vortex veins in 1 or 2 quadrants of the eyes of C57BL/6 mice. In these mice, we observed focal choroidal thickening only around the sutured vortex veins in the cryosections obtained on days 1 and 2 (S2 Fig). Therefore, herein we sutured all vortex veins to induce choroidal congestion in the eye (Fig 1A and 1B). We first treated BALB/c mice, allowing choroidal vessels to be seen by fundus examination because of the non-pigmented RPE. We were able to confirm the dilated choroidal vessels due to vortex vein congestion, despite the retinal vessels being unchanged on day 1 (Fig 2A and 2B). Next, we conducted ultra-widefield ICGA in C57BL/6 mice 1 day after suturing the vortex veins. The vortex veins were dilated from the posterior pole to the ampulla of the vortex veins in the treated eyes (Fig 2D), as compared to the control eyes (Fig 2C).

**Fig 3:**
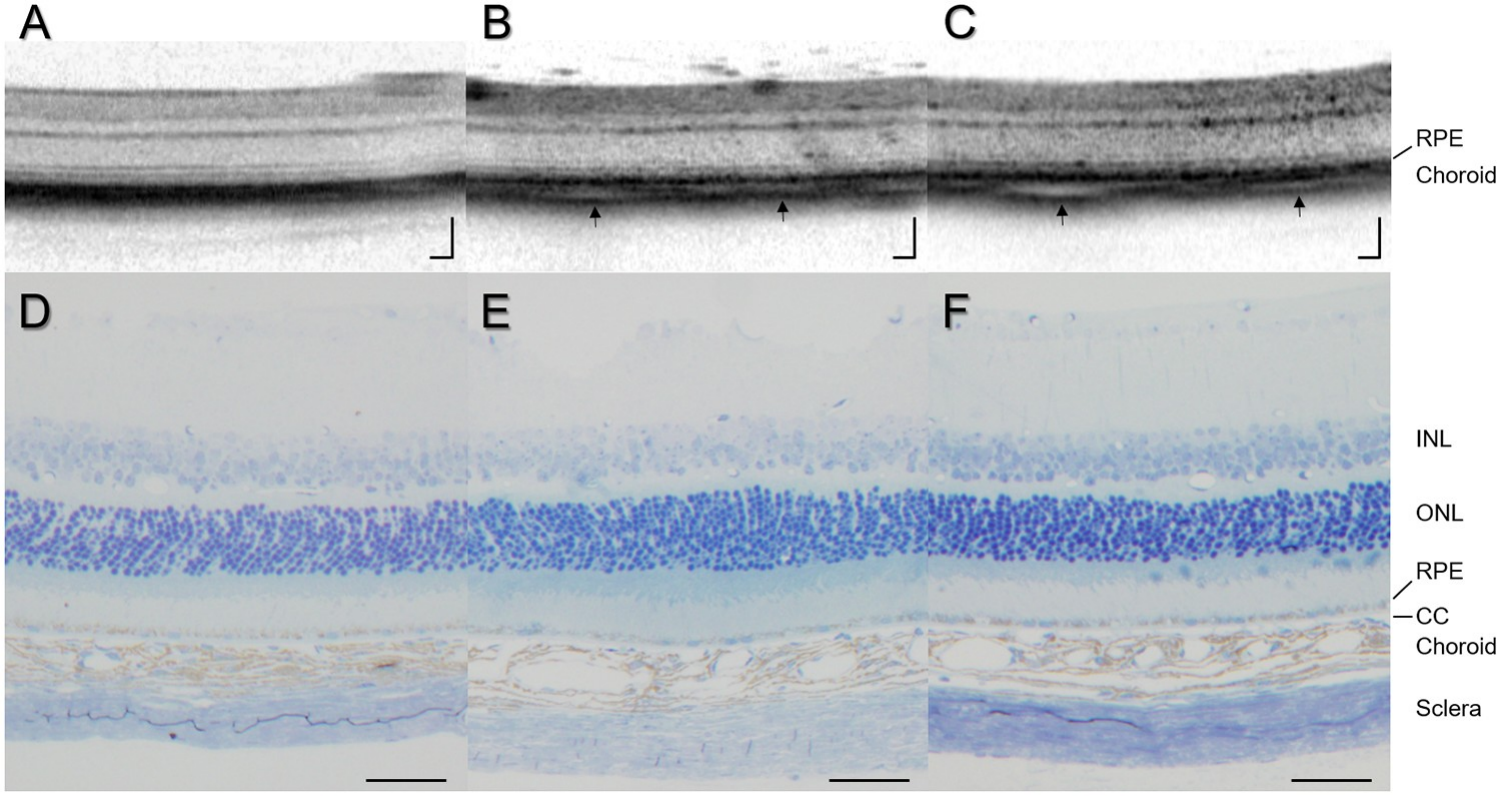
Choroidal thickening associated with dilated choroidal vessels after induction of choroidal congestion. (A) OCT shows normal retinochoroidal structure in the C57BL/6 mouse control eye. (B and C) 1 and 7 days after suturing of the vortex veins, thickened choroid with areas of low intensity is noted (black arrows). (D) Resin-embedded section of C57BL/6 mouse control eye shows normal retinochoroidal structure. (E and F) 1 and 7 days after suturing of the vortex veins, thickened choroid associated with dilated choroidal vessels is observed despite the normal retinal structure. The choriocapillaris layer appearance looks unchanged. RPE: retinal pigment epithelium, INL: inner nuclear layer, ONL: outer nuclear layer, CC: choriocapillaris, scale bar: 100μm (A-C), 50μm (D-F).

Then, we performed OCT on the C57BL/6 mice with induction of choroidal congestion. The treated eyes showed thickened choroid on days 1 and 7 (Fig 3B and 3C) as compared to the control eyes (Fig 3A). The OCT images of the treated eyes showed low intensity areas in the choroid, which corresponded to the dilated choroidal vessels (Fig 3B and 3C). We also examined resin-embedded sections of C57BL/6 mouse eyes 1 and 7 days after induction of choroidal congestion. The choroidal thickness was greater in the treated eyes (Fig 3E and 3F) than in the control eyes (Fig 3D). The eyes with induction of choroidal congestion showed thickened choroid with dilatation of choroidal vessels whereas the retina appeared normal (Fig 3E and 3F). These features are consistent with the OCT findings. The choriocapillaris layer on resin-embedded sections looked unchanged 1 and 7 days after suturing of the vortex veins.

**Fig 4:**
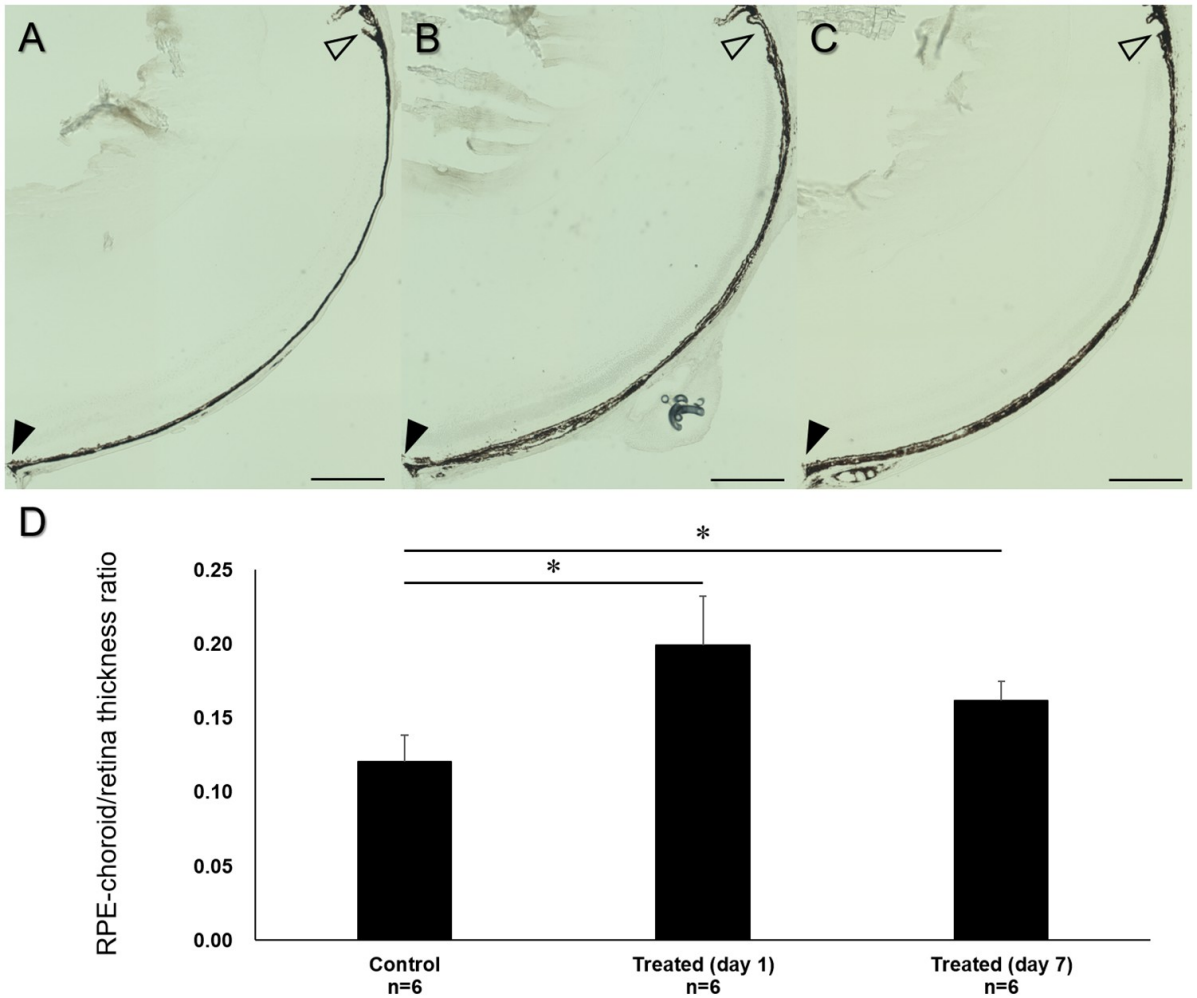
Quantitative evaluation of choroidal thickening after suturing of the vortex veins. (A) Cryosection of C57BL/6 mouse control eye shows thin choroid (black arrowhead: margin of optic nerve, empty arrowhead: ciliary body). (B and C) 1 and 7 days after suturing of the vortex veins, thickened choroid is observed from the posterior pole to the ciliary body. (D) The RPE-choroid/retina thickness ratio at 600μm from the margin of the optic nerve was 0.12±0.02 in the control group, 0.19±0.03 in the treated day 1 group, and 0.16±0.01 in the treated day 7 group (n = 6 each). The ratios in the treated day 1 and 7 groups were significantly greater than that in the control group (*P<0.05 each). Scale bar: 300μm (A-C).

We further examined cryosections of C57BL/6 mouse eyes 1 and 7 days after suturing of the vortex veins. To quantitatively evaluate the choroidal thickening after the induction of choroidal congestion, we calculated the RPE-choroid/retina thickness ratios in 6 eyes each of the control, treated day 1, and treated day 7 groups. The RPE-choroid/retina thickness ratios were 0.12±0.02, 0.19±0.03, and 0.16±0.01, respectively. The ratios in the treated day 1 and day 7 eyes were significantly greater than that in the control eyes (P<0.05 each, Fig 4).

Next, we examined gene expressions by microarray analysis using the RPE–choroid–sclera complex of eyes from mice 1 day after choroidal congestion had been induced. The RNAs of inflammatory response factors including *Cxcl3*, *Il6*, *Ccl24*, *Ccl7*, *Ccl2*, and *Il1b* were upregulated in treated eyes as compared to control eyes (Table 1). On the other hand, *VEGF-A* and *PlGF* were not upregulated in the treated eyes (Table 1).

**Table 1:** DNA microarray analysis using the retinal pigment epithelium–choroid–sclera complex.

Gene Name	Gene Symbol	Fold Change (Treated day 1:Control)
chemokine (C-X-C motif) ligand 3	Cxcl3	423
interleukin 6	Il6	227
chemokine (C-C motif) ligand 24	Ccl24	137
chemokine (C-C motif) ligand 7	Ccl7	115
chemokine (C-C motif) ligand 2	Ccl2	108
interleukin 1 beta	Il1b	98
placental growth factor	PlGF	-2
vascular endothelial growth factor A	VEGF-A	within±1.5

RNAs of inflammatory response factors including *Cxcl3*, *Il6*, *Ccl24*, *Ccl7*, *Ccl2*, and *Il1b* were upregulated in treated day 1 eyes as compared to control eyes, whereas RNAs of angiogenic factors such as *VEGF-A* and *PlGF* were not upregulated in the treated eyes.

**Fig 5:**
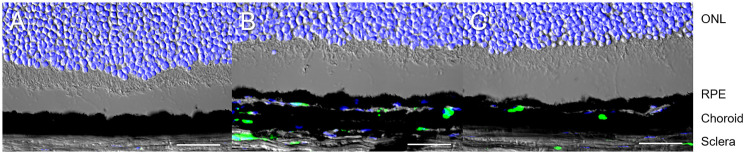
Migration of macrophages into the choroid after induction of choroidal congestion. (A) Immunohistochemistry using anti-CD11b antibody for control C57BL/6 mouse eye shows essentially no CD11b positive cells in either the retina or the choroid. (B) 1 and 7 days after vortex vein suturing, CD11b positive cells are detected in the thickened choroid, indicating the macrophages migrating into the choroid. DAPI: blue, CD11b: green, ONL: outer nuclear layer, RPE: retinal pigment epithelium, scale bar: 30μm.

To further investigate the inflammatory response, we performed immunohistochemistry using anti-CD11b antibody on cryosections to detect macrophages migrating into the eye. This is because microarray analysis revealed the upregulated *Ccl2* expression in the treated eyes. CD11b positive cells were observed in the thickened choroid on day 1 and 7 in the treated eyes (Fig 5B and 5C), whereas almost no CD11b positive cells were detected in the control eyes (Fig 5A).

**Fig 6:**
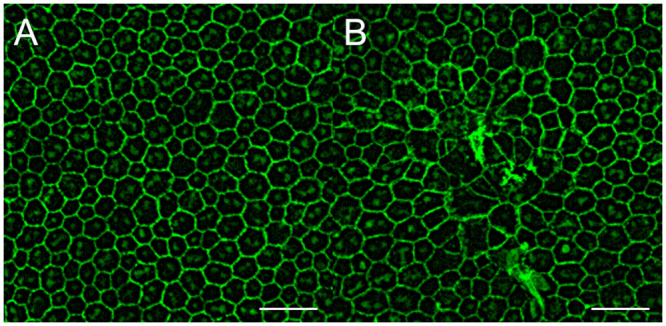
Alterations of the retinal pigment epithelium after suturing of the vortex veins. (A) Immunohistochemistry using anti-ZO-1 antibody for RPE flatmount in the control C57BL/6 mouse eye shows well organized and packed RPE cells. (B) 7 days after the vortex vein suturing, focal RPE cell degeneration is observed. Scale bar: 50μm.

Finally, we investigated the morphological changes in RPE cells by immunohistochemistry using anti-ZO-1 antibody on RPE flatmount of C57BL/6 mouse eyes. Interestingly, focal RPE cell degeneration was observed 7 days after choroidal congestion had been induced in these eyes (Fig 6A and 6B).

## Discussion

We attempted to establish a mouse model of pachychoroid by suturing vortex veins at the surface of the sclera. The choroidal thickening associated with dilatation of choroidal vessels was observed after establishing vortex vein congestion. Moreover, microarray analysis using the RPE–choroid–sclera complex and immunohistochemistry revealed upregulation of the expressions of inflammatory response-related RNAs and the migration of macrophages into the choroid after suturing of the vortex veins, respectively. We also observed the RPE degeneration in this choroidal congestion mouse model.

Nishikawa et al. created a monkey model of choroidal circulatory disturbance by suturing and cauterizing 2 vortex veins at the site of exit from the sclera [26]. They evaluated ICGA findings immediately after the occlusion and identified correlations with the histopathological changes. ICGA showed delayed filling of choroidal arteries in the area of occluded vortex veins, pulsatile retrograde filling of the choroidal veins, increased intensity of fluorescence in the larger veins, and delayed clearance of the indocyanine green dye [26]. Moreover, their histopathological analysis revealed the choroidal vessels to be vertically dilated after the occlusion [26]. These findings are present in eyes with pachychoroid spectrum diseases. Therefore, this study supports the concept that congestion of vortex veins might be related to the development of pachychoroid spectrum diseases. Hayreh cauterized 1, 2, 3, or all 4 vortex veins in monkey eyes and evaluated the short- and long-term effects on the eyes [27]. Fluorescein angiography showed filling of the choroidal vascular bed to be sluggish and delayed in the short term [27]. However, these changes were reversed in the long term. He reported the exact mechanism of normalization, i.e. the restoration of the vascular bed, to be unknown but suggested collateral formation as a possible compensatory mechanism for the occlusion of vortex veins [27]. In the current study, the choroid was significantly thickened after suturing of the vortex veins, whereas the choroidal thickness on day 7 was slightly less than that on day 1. The choroidal congestion might become milder on day 7 than on day 1, probably due to collateral formation as well as the recanalization of occluded vortex veins.

Pro-inflammatory cytokines such as IL-6 and MCP-1 were reportedly detected in the aqueous humor from patients with CSC and PNV [14, 15]. Thus, it has been suggested that inflammation might be related to the pathological mechanism of pachychoroid spectrum diseases. The microarray analysis using the RPE–choroid–sclera complex revealed that expressions of the RNAs related to the inflammatory response, including *Il6* and *Ccl2*, were upregulated in our choroidal congestion model on day 1. Therefore, our choroidal congestion mouse model might reflect pachychoroid spectrum diseases in terms of the underlying molecular mechanisms. Previous studies found that the concentrations of VEGF-A and PlGF in aqueous humor were upregulated, with the level approaching statistical significance along with disease progression from acute to chronic CSC, whereas the upregulation was not significant from chronic CSC to PNV [15]. In this study, expressions of angiogenesis related RNAs, including *VEGF* and *PlGF*, were not upregulated on day 1. Expressions of the RNAs of molecules related to angiogenesis might be upregulated in the long term after induction of choroidal congestion.

In the current study, RPE degeneration was observed on day 7 in our mouse model of choroidal congestion, a finding similar to RPE atrophy detected in patients with PPE [3]. Thinning of the inner choroid including the choriocapillaris has reportedly been attributed to dilatation of the outer choroidal vessels, leading to RPE atrophy in eyes with PPE. However, resin-embedded sections from days 1 and 7 of our choroidal congestion mouse model showed neither occlusion nor thinning of the choriocapillaris. These results suggest that RPE degeneration due to congestion of the choroidal circulation might occur first, followed by the inner choroid including the choriocapillaris possibly becoming thinner due to reduced VEGF secretion from degenerated RPE cells as a secondary change. Further long-term studies are needed to elucidate the relationship between RPE degeneration and the choriocapillaris changes in our mouse model of choroidal congestion.

In this study, we evaluated the acute phase of our choroidal congestion mouse model. However, pachychoroid spectrum diseases are thought to be chronic in nature. Therefore, chronic changes including occlusion of the choriocapillaris, development of serous retinal detachment due to disruption of the outer blood-retinal barrier, and development of choroidal neovascularization should be assessed in this mouse model of choroidal congestion.

In conclusion, we have established a choroidal congestion mouse model by suturing of the vortex veins. This model shows some features observed in pachychoroid spectrum diseases. Although the choroidal structures, including vessel density and thickness, differ between human and mouse eyes, this mouse model is potentially useful for investigating the pathophysiology of pachychoroid spectrum diseases and thereby devising novel treatments.

## Supporting information

S1 Video Surgical procedure for the induction of choroidal congestion in mouse eye. (MP4)

S1 Fig Measurement of RPE-choroid/retina thickness ratio. The RPE-choroid/retina thickness ratio was measured on the cryosection including the center of optic nerve. The ratio was measured at 600μm from the margin of the optic nerve (red dashed line: 600μm from the margin of optic nerve, black line: retinal thickness, white line: RPE-choroidal thickness).
(TIF)

S2 Fig Cryosection from preliminary experiments. 1 day after suturing 1 vortex vein in C57BL/6 mouse eye, a slightly thickened choroid is observed at the quadrant in which the vortex vein was sutured (black arrowhead). Scale bar: 500μm.
(TIF)
