# Supplementary figures and images for: Choroidal congestion mouse model: Could it serve as a pachychoroid model?
Source: PLoS One. 2021 Jan 28;16(1):e0246115. doi: 10.1371/journal.pone.0246115 (PMC7843010; doi:10.1371/journal.pone.0246115)

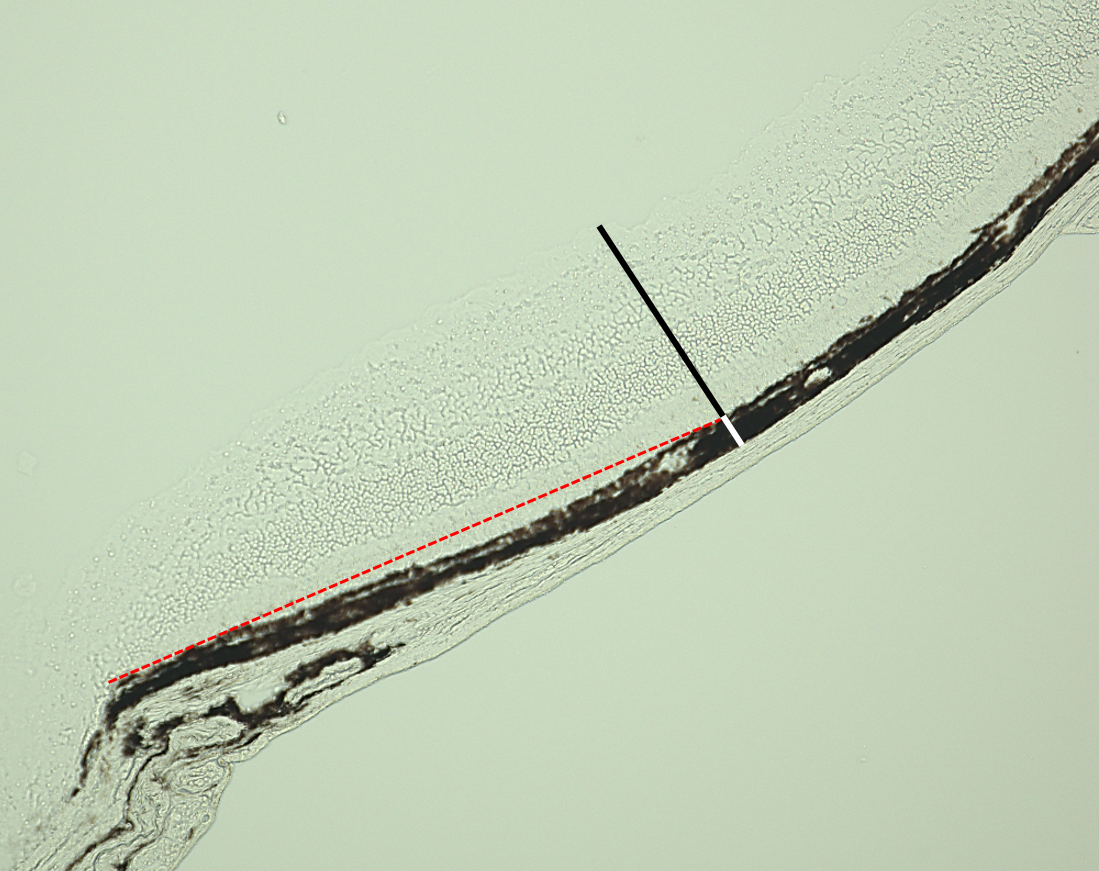

Supplement: S1 Fig — The RPE-choroid/retina thickness ratio was measured on the cryosection including the center of optic nerve. The ratio was measured at 600μm from the margin of the optic nerve (red dashed line: 600μm from the margin of optic nerve, black line: retinal thickness, white line: RPE-choroidal thickness). (TIF) [file pone.0246115.s002.tif]

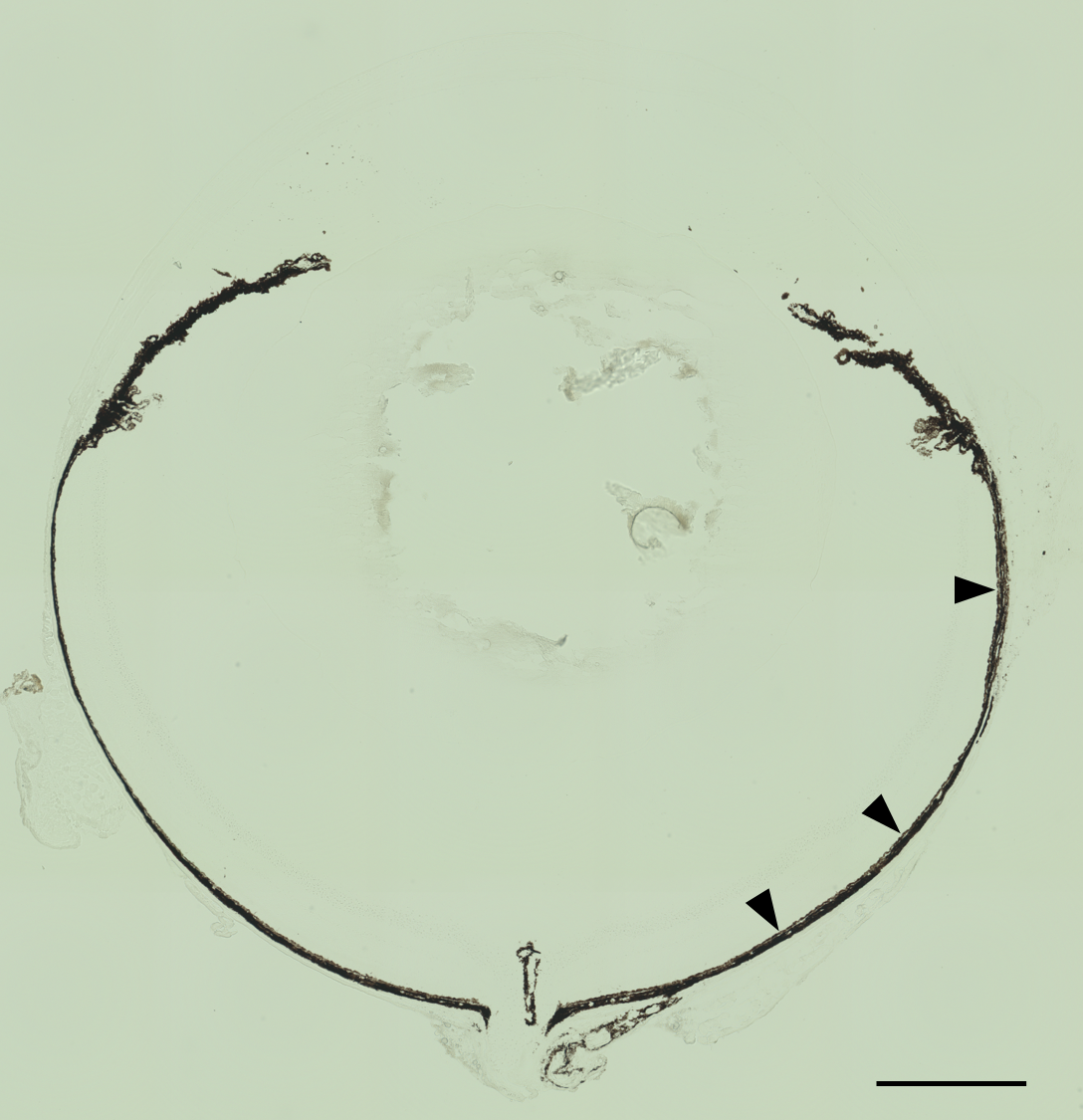

Supplement: S2 Fig — 1 day after suturing 1 vortex vein in C57BL/6 mouse eye, a slightly thickened choroid is observed at the quadrant in which the vortex vein was sutured (black arrowhead). Scale bar: 500μm. (TIF) [file pone.0246115.s003.tif]
